# Genome Improvement and Core Gene Set Refinement of *Fugacium kawagutii*

**DOI:** 10.3390/microorganisms8010102

**Published:** 2020-01-11

**Authors:** Tangcheng Li, Liying Yu, Bo Song, Yue Song, Ling Li, Xin Lin, Senjie Lin

**Affiliations:** 1 State Key Laboratory of Marine Environmental Science and College of Ocean and Earth Sciences, Xiamen University, Xiamen 361102, China; tangcheng.li86@gmail.com (T.L.); yly20070567@126.com (L.Y.); lingli@xmu.edu.cn (L.L.); 2 Department of Marine Sciences, University of Connecticut, Groton, CT 06340, USA; 3 Agricultural Genomics Institute at Shenzhen, Chinese Academy of Agricultural Sciences, Shenzhen 518124, China; songbo446@yeah.net; 4 BGI-Qingdao, BGI-Shenzhen, Qingdao 266555, China; songyue@genomics.cn; 5 Laboratory of Marine Biology and Biotechnology, Qingdao National Laboratory of Marine Science and Technology, Qingdao 266237, China

**Keywords:** RNA-seq, Symbiodiniaceae, *Fugacium kawagutii*, gene set, genome, core genes, Hi-C

## Abstract

Cataloging an accurate functional gene set for the Symbiodiniaceae species is crucial for addressing biological questions of dinoflagellate symbiosis with corals and other invertebrates. To improve the gene models of *Fugacium kawagutii*, we conducted high-throughput chromosome conformation capture (Hi-C) for the genome and Illumina combined with PacBio sequencing for the transcriptome to achieve a new genome assembly and gene prediction. A 0.937-Gbp assembly of *F. kawagutii* were obtained, with a N50 > 13 Mbp and the longest scaffold of 121 Mbp capped with telomere motif at both ends. Gene annotation produced 45,192 protein-coding genes, among which, 11,984 are new compared to previous versions of the genome. The newly identified genes are mainly enriched in 38 KEGG pathways including *N*-Glycan biosynthesis, mRNA surveillance pathway, cell cycle, autophagy, mitophagy, and fatty acid synthesis, which are important for symbiosis, nutrition, and reproduction. The newly identified genes also included those encoding *O*-methyltransferase (*O*-MT), 3-dehydroquinate synthase, homologous-pairing protein 2-like (HOP2) and meiosis protein 2 (MEI2), which function in mycosporine-like amino acids (MAAs) biosynthesis and sexual reproduction, respectively. The improved version of the gene set (Fugka_Geneset _V3) raised transcriptomic read mapping rate from 33% to 54% and BUSCO match from 29% to 55%. Further differential gene expression analysis yielded a set of stably expressed genes under variable trace metal conditions, of which 115 with annotated functions have recently been found to be stably expressed under three other conditions, thus further developing the “core gene set” of *F. kawagutii*. This improved genome will prove useful for future Symbiodiniaceae transcriptomic, gene structure, and gene expression studies, and the refined “core gene set” will be a valuable resource from which to develop reference genes for gene expression studies.

## 1. Introduction

*Fugacium kawagutii* is one of the Symbiodiniaceae dinoflagellates, which are mostly essential endosymbionts of reef corals and many other invertebrates [1]. The mutualistic relationship between Symbiodiniaceae and corals as well as other invertebrates is foundational to the existence of the tropical and subtropical coral reef ecosystem and the biodiversity paradise in the world’s ocean. In the mutualistic symbiosis, a symbiotic Symbiodiniaceae provides its coral host indispensable organic nutrients and oxygen, in exchange, the coral host provides inorganic nutrients, carbon dioxide, and serves as shelter for the symbiont [2,3]. The intricate coral-Symbiodiniaceae symbiosis, however, is susceptible to global climate change and anthropogenic disturbances, which have been blamed for the massive coral bleaching and degradation that have caused rapid loss of the coral reef in the past decades [4]. Coral bleaching has been attributed to the disruption of the symbiosis and expulsion of the symbionts triggered by the natural and anthropogenic environmental stresses [5,6]. Our understanding of the biological mechanism and its response to the environmental insults is still limited, to a large extent because there is a dearth of genomic and functional genetic information in both the coral host and the Symbiodiniaceae endosymbionts. Recent years have witnessed a rapid increase in high-throughput genome, transcriptome sequencing, and significant enhancement of molecular understanding of the symbiosis, while current genome data (genome and gene models) are still with quality compromises due to the complexities of the genome architecture, especially for the immense dinoflagellate genomes.

The Symbiodiniaceae family has nine genera [7], among which, all but *Effrenium* contain endosymbionts. So far, six species from four of the genera have been subjected to genome sequencing: *Breviolum minutum* [8], *Fugacium kawagutii* [9,10], *Symbiodinium microadriacticum* [11], *Cladocopium goreaui* [10], *Symbiodinium* spp., and *Cladocopium* spp. [12]. These studies have assembled 41.07% to 88.98% of their respective genomes, predicting 26,609–69,018 protein coding genes. These genome assemblies, however, are not complete, and still have room for refinement. For *F. kawagutii*, for instance, a recent repeated sequencing [10] provided improvement to the original assembly [9], illustrating the need for continued effort to refine the genome data.

*F. kawagutii* (formerly *Symbiodinium kawagutii*, Clade F) was originally isolated from the scleractinian coral *Montipora verrucosa* in Hawaii, where ambient temperature is about 25 °C [1]. Previous research found that *F. kawagutii* can hold a stable maximum quantum yield of PSII (Fv/Fm) and a growth rate under higher temperatures than other species, such as *Cladocopium* spp. [13,14]. This makes *F. kawagutii* a potential thermal-resistant species that confers heat resistance to its hosts, as is the case for *Durusdinium* [15,16]. Therefore, *F. kawagutii* is an important species to study. To improve genome annotation, high-throughput cDNA sequencing (RNA-Seq) technologies have been used to correct gene structure [17], detect new alternative splicing isoforms [18], and discover new genes and new transcripts [19,20]. In this study, we updated and refined the genome data of the *F. kawagutii* using high-throughput chromosome conformation capture (Hi-C) on the genome and Illumina combined with PacBio sequencing on transcriptomes. Our results led to significant improvement of the *F. kawagutii* genome and protein-coding gene repertoire. In addition, we screened for genes that showed no significant changes in expression under different treatment conditions, and further developed the recently initiated “core gene set” of *F. kawagutii*, a valuable resource for developing reference genes for gene expression studies.

## 2. Materials and Methods

### 2.1. Algal Culture and Trace Metal Treatments

*F. kawagutii* (strain CCMP2468) was provided by the National Center for Marine Algae and Microbiota, and was grown in seawater medium at 26 °C under a 12 h:12 h light:dark cycle with 680 μmol quanta m^−2^ s^−1^ photon flux. L1 medium [21] was used with slightly modified trace metal concentrations and prepared in surface seawater collected from the South China Sea. For trace metal-deficient treatment methods, refer to those previously reported [22,23]. Briefly, the control cultures were supplied with 1.25 nM Fe′, 0.5 pM Cu′, 4.2 nM Mn′, 6.7 pM Ni′, and 12.5 pM Zn′ upon addition of 20 μM ethylenediaminetetraacetic acid (EDTA). Trace metal-deficient treatment groups consisted of three treatments lacking either Cu, Mn, or Ni (denoted as –Cu, –Mn, and –Ni, respectively) and two treatments with a one fifth concentration of Zn and Fe (denoted as + 1/5 Zn and + 1/5 Fe, respectively) of that in the control.

### 2.2. RNA Extraction

Approximately 10^7^ cells of each sample were harvested for RNA extraction. Total RNA was isolated following a previously reported method [24] with slight modification. Briefly, cells were homogenized thoroughly using the Fastprep^®^-24 Sample Preparation System (MP Biomedicals, LLC, Santa Ana, CA, USA) with beads (0.5 mm mixed 0.1 mm diameter ceramic beads at 3:1), run for 3 cycles each at a speed of 6 M/s for 1 min. RNA was isolated using Trizol reagent (Molecular Research Center, Inc., Cincinnati, OH, USA) coupled with further purification using Direct-Zol RNA Miniprep (Zymo Research, Orange, CA, USA). The quality and the concentration of the extracted RNA were measured using an Agilent 2100 Bioanalyzer (Agilent Technologies, Palo Alto, CA, USA) and NanoDrop (ND-2000 spectrophotometer; Thermo Scientific, Wilmington, DE, USA).

### 2.3. Hi-C, Illumina RNA-Seq, and PacBio Sequencing

Hi-C libraries was constructed according to a protocol on protocol.io [25]. Briefly, approximately 10^7^ cells were collected and fixed in formaldehyde, from which nuclei were isolated. Endonuclease (*Dpn* II) was then added and the nucleus sample was incubated at 62 °C for 20 min to digest DNA, followed by the incubation with a mixture of Klenow fragment, biotin-labelled dCTP, dTTP, dATP, and dGTP. After the ligation of fragmented DNA, proteinase K was added and the solution was incubated for 30 min. Then, the remaining DNA was precipitated and the biotin-labeled fragments were selected for library construction. Two Hi-C libraries were constructed and sequenced using the BGISEQ-500 platform. A total of 260 Gb raw data was produced.

To achieve a cost-effective deep sequencing of the transcriptome, an equal aliquot of RNA from each of the nutrient conditions (nutrient-replete, –Cu, –Mn, –Ni, + 1/5 Zn, and + 1/5 Fe) were combined to produce a RNA mixture > 5 μg, which was subjected to Illumina RNA-Seq sequencing (10 Gbp) and PacBio single-molecule sequencing (15 Gbp) using HiSeq × ten platform and PacBio ISO-Seq platform (BGI Genomics Co., Ltd.), respectively. All raw sequencing reads were deposited in the SRA database under Accession No. SAMN13258281. Illumina RNA-Seq sequencing raw reads were subject to quality filtering by removing sequences that contain adapters, >10% unknown nucleotides, or >50% of low quality (Q-value ≤ 10) bases. PacBio single-molecule sequencing reads were filtered to obtain high-quality full-length transcripts according to single molecular real-time (SMRT) link v5.0.1 pipeline provided by Pacific Bioscience [26]. Then, we used Illumina RNA-Seq reads to further correct the PacBio single-molecule reads by using Proovread software [27].

### 2.4. Genome Improvement and Gene Set Refinement

To improve genome assembly, the 260 Gb Hi-C data was along with our original scaffolds [9]. We first used HiC-Pro software (version2.8.0_devel) [28] with default parameters to get ~26 Gb valid sequencing data. Then, Juicer (version 1.5) [29], and the three-dimensional (3D) de novo assembly pipeline [30] were used to connect the scaffolds to chromosomes or chromosome-level super-scaffolds.

Based on the two high-quality sequencing datasets described above, the protein-coding gene set of the *F. kawagutii* genome was refined following the GETA gene annotation method (https://github.com/chenlianfu/geta), which combines RNA-aided annotation, homology searches, and de novo prediction. Firstly, repeat-masked genome assembly was obtained by using RepeatMasker based on repeat sequences identified with RepeatModeler. Secondly, the next-generation clean reads were aligned to the genome sequences using HISTA2 [31], and then genes were predicted based on the open reading frame (ORF) of the optimal transcripts. Thirdly, homologous annotation was conducted by searching *F. kawagutii* genome scaffolds against a local database containing Pacbio-sequences and dinoflagellate protein sequences from UniPort database with BLAST, followed by Genewise annotation. Fourthly, de novo annotation was performed using AUGUSTUS. Finally, the above three gene annotations were integrated to obtain the final result (Fugka_Geneset_V3).

Fugka_Geneset_V3 was functionally annotated using seven reference databases (NCBI non-redundant protein database (Nr), NCBI non-redundant nucleotide database (Nt), Swissprot, Kyoto Encyclopedia of Genes and Genomes (KEGG), Eukaryotic Orthologous Groups (KOG), Pfam, and Gene Ontology (GO)). The Benchmarking Universal Single-Copy Orthologs (BUSCO) [32] was used to evaluate gene completeness. The Fugka_Geneset_V3 and annotation were available in the Symbiodiniaceae and Algal Genomic Resource database (SAGER, http://sampgr.org.cn).

### 2.5. Core Gene Set Update

To investigate differential gene expression patterns under the different trace metal conditions, the RNA samples of *F. kawagutii* from nutrient-replete, –Cu, –Mn, –Ni, + 1/5 Zn, and + 1/5 Fe conditions were also separately sequenced using BGI RNA-Seq [23]. Genes commonly and highly expressed across all six treatment conditions were identified as core genes, following previously reported methods [33]. Briefly, these were genes showing expression of ≥ 90% average Transcripts Per Million (TPM) and coefficient of variation (CV) ≤ 0.08 across the six treatment conditions. Then, cores genes selected from nutrient replete and trace metal deficiencies were used to compare with the previous core gene set [33] using BLASTN with *E*-value ≤ 10^−5^ to generate a new core gene set.

### 2.6. Validation of Reference Genes for Reverse Transcription Quantitative PCR (RT-qPCR)

Core genes are likely essential genes for the species to sustain growth under any conditions, and are thus candidates for reference genes in gene expression studies. In this study, we assessed seven genes from “core gene set” for their suitability as reference genes in *F. kawagutii*. For each sample, 400 ng total RNA was used in cDNA synthesis using PrimeScript^TM^ RT reagent Kit (Takara, Clontech, Japan) that contained the genomic erase buffer. Based on the gene fragment obtained from Fugka_Geneset_V3, specific primers were designed and synthesized (Supplementary Table S1). Reverse transcription quantitative PCR (RT-qPCR) was performed using iTaq^TM^ Universal SYBR^®^ Green Supermix on a CFX96 real-time PCR System (Bio-Rad Laboratories, Hercules, USA) and the reaction was carried out in a total volume of 12 μL containing 2.5 µM of each primer, cDNA equivalent to 5 ng of total RNA, and 6 µL Supermix [34]. Stability of gene expression was evaluated using geNorm [35].

## 3. Results and Discussion

### 3.1. Genome Improvement and Gene Set Refinement

With the advance in high-throughput sequencing (HTS), six draft genomes from four genera (*Symbiodinium*, *Breviolum*, *Cladocopium,* and *Fugacium*) have been sequenced. These genomes have assembly rates ranging from 41.07% to 88.98% and rates at which genes are supported by expressed sequencing tag (EST) ranging from 62.5% to 77.2% (Table 1). The *F. kawagutii* draft genome was first generated in 2015 [9] and later improved in 2018 [10], with high-quality gene models resulting from both (Fugka_Geneset_V1 and Fugka_Geneset_V2). With either version of the genome, however, only 25% to 33% of our transcriptomic reads generated in this study can be mapped to the gene models, indicating that the gene models need to be further refined and additional genes to be identified (Table 2). In this study, we improved genome assembly with Hi-C data, and refined the set of gene models based on the high-quality mRNA sequences from the PacBio ISO-Seq platform (for long read length) and the Illumina HiSeq × ten platform (for accurate sequencing reads).

**Figure 1:**
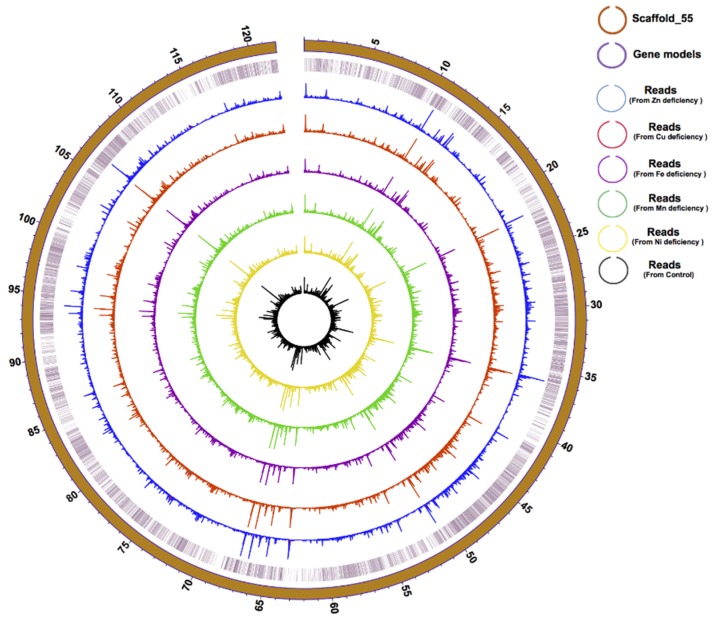
Physical distribution and expression profile of genes in the longest (121 Mbp) Scaffold in *F. kawagutii.* The circles from the outside to the inside mark scaffold length (in Mbp), gene location, and gene expression (bar height) under Zn-, Cu-, Fe-, Mn-, and Ni-deficient as well as normal conditions.

The integration of our Hi-C and transcriptomic data resulted in a 0.937 Gbp genome assembly of the *F. kawagutii* genome, constituting 29,213 scaffolds, with scaffold N50 of 13,533,496 bp compared to 380,908 bp in version 1 (Supplementary Table S2). In this significantly improved assembly, the longest scaffold (scafflold_55) is 121 Mbp in length (Figure 1) and contains telomeric motif (TTTAGGG) at both ends, indicating a chromosome-level assembly.

Gene structure prediction yielded 45,192 gene models, with an average transcript length of 1679 bp and an N50 of 1,950 bp. All coding sequences have a relatively high G + C content (54%), similar to other Symbiodiniaceae species examined so far (57.7% in *S. microadriaticum*, 52.7% in *B. minutum*, 56.67% in *C. goreaui*). Of the 45,192 genes, 39,885 (88.26%) were annotated in the Nr database, 16,580 (36.7%) were annotated in the KEGG database, and 7683 (17.00%) were annotated in the GO database, producing a total of 42,796 (94.70%) genes functionally annotated to all seven reference databases. BUSCO analysis gave a 55% match rate for the Fugka_Geneset_V3 database, a significant improvement from the previous 29.2% for Fugka_Geneset_V1 and 45% for Fugka_Geneset_V2 (Table 3). Furthermore, 41,080 gene models (90.09%) in Fugka_Geneset_V3 were supported by EST (Table 1). Genes predicted for the longest scaffold were highly supported by expressed genes from six of our transcriptomes (Figure 1).

Both Fugka_Geneset_V1 and Fugka_Geneset_V2 mainly relied on the reads from the next-generation sequencing (NGS) to predict the coding sequences (CDS). The remarkable improvement in Fugka_Geneset_V3 clearly has benefited from PacBio single-molecule sequencing [26], which facilitates mapping unigenes to the genome and generating accurate CDS. Despite the increase in the RNA-Seq read mapping rate from 33% to 54%, there is still about 15% of the transcriptome sequencing reads that could not be mapped to Fugka_Geneset_V3, although they did map to the genome (Table 2). There are several possible explanations. Firstly, there might be protein-coding genes that were not predicted in Fugka_Geneset_V3, because the gene finding algorithm we used has not been optimized for dinoflagellate genomes. Secondly, the RNA-Seq reads might also contain the reads of non-coding genes, e.g., long noncoding RNA (lncRNA). Thirdly, non-canonical transcriptions might make the mature mRNA not match to the genome, e.g., alternative transcriptions [36], delRNAs [37,38]. To find out the exact reason, efforts to further understand the genome structure and gene transcription mechanisms of *F. kawagutii* are needed.

### 3.2. Characterization of Newly Identified Genes

**Figure 2:**
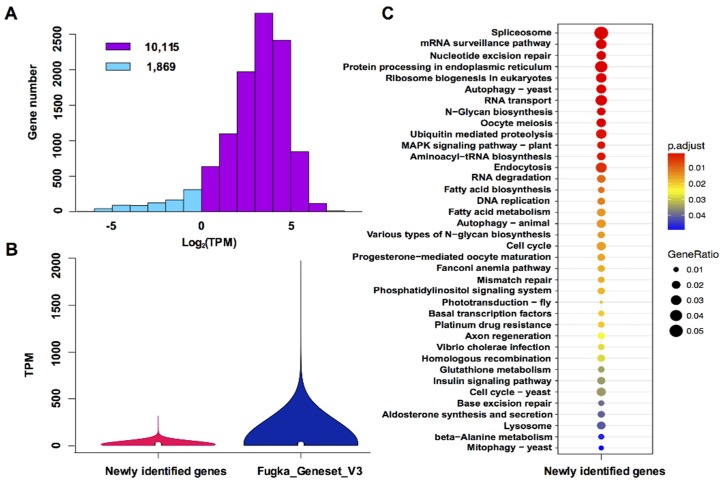
Expression of newly identified genes and significantly enriched pathways: (**A**) Bar chart of gene expression. Blue, transcripts per million (TPM) < 1; pink, TPM > 1. (**B**) Comparison of expression profiles between newly identified genes and all genes in Fugka_Geneset_V3. (**C**) Significantly enriched KEGG pathways of newly identified genes (adjust *p*-value < 0.05).

To mesh truly new genes, we compared Fugka_Geneset_V3 to Fugka_Geneset_V1 and Fugka_Geneset_V2 by using BLASTN (*E*-value ≤ 10^−5^) and removed shared orthologous groups. We found 11,984 genes which showed no base coverage with previously reported genes (Supplementary Table S3). Of these genes, 11,211 were detected in the RNA-Seq database from five trace metal deficiencies and 10,113 of them were expressed at TPM > 1 under our experimental conditions (Figure 2A). Further TPM comparison revealed that the overall expression of the newly identified genes was lower than the average expression level of Fugka_Geneset_V3 genes (Figure 2B). The lower expression levels might explain why they were not identified previously. Of the 11,984 newly identified genes, 3241 (27%) were mainly enriched in 38 KEGG pathways (Figure 2C), including mRNA surveillance, N-glycan biosynthesis, cell cycle, and fatty acid synthesis (Figure 2C). From these significantly enriched KEGG pathways, two themes emerged.

The first emergent theme is biosynthesis of amino acids and N-glycan biosynthesis. Corals lack the capacity to synthesize some amino acids, e.g., cysteine and tryptophan in *Acropora digitifera* [39], thus the ability to synthesize these amino acids makes the Symbiodiniaceae functionally complementary to, and hence, potentially compatible symbionts of the coral. In the present study, three genes encoding cysteine synthase and one gene encoding tryptophan synthase were found from the newly identified genes (Supplementary Table S4), and their sequences were completely different from previously reported homologs in Fugka_Geneset_V1 and Fugka_Geneset_V2. Mycosporine-like amino acids (MAAs) act as antioxidants scavenging reactive oxygen species (ROS) in coral and other marine organisms. The four major MAAs biosynthesis enzymes, dehydroquinate synthase (DHQS), *O*-methyltransferase (*O*-MT), ATP-grasp, and non-ribosomal peptide synthetase (NRPS), were missing in Fugka_Geneset_V1 [9]. In the present study, one gene encoding *O*-MT and two genes encoding 3-dehydroquinate synthase are present in the newly identified genes set (Supplementary Table S5). These enzymes were also reported previously in Fugka_Geneset_V2, suggesting that MAA biosynthesis was not lost in *F. kawagutii* but rather was missed in the original genome gene models [9]. If production of MAA is demonstrated, this will challenge the long-held notion that only clade A Symbiodiniaceae has retained the ability to produce MAAs [40,41]. Furthermore, glycoproteins in mutualistic Symbiodiniaceae play important roles in conjunction with a host-associated pattern recognition receptor, which mediates recognition by a host [42]. The various types of N-glycan biosynthesis were enriched in the KEGG pathways of our newly identified genes (Figure 2C), but as in our previous research [9], four genes (*MAN2*, *MGAT2*, *MGAT4*, *MGAT5*) in the conventional glycan biosynthesis are missing.

The second emergent theme is the functions related to cell cycle and sexual reproduction. Based on population genetic data and inventory of meiotic genes, it has been postulated that Symbiodiniaceae can reproduce sexually and have a diploid life stage when exposed to stressful conditions [43,44,45]. A previous study [10] found 46 predicted meiosis proteins of the 51 toolkit (*E* ≤ 10^−5^) in *F. kawagutii*, while three of the eleven meiosis-specific proteins were not detected in *F. kawagutii* [10]. Here, we found one of these, homologous-pairing protein 2 (*HOP2*), in our newly identified genes set. MEI2-like genes comprise a five-member gene family, which play a role in meiosis and vegetative growth in *Arabidopsis* [46]. Multiple MEI2-like genes have been documented in the previous assemblies of *F. kawagutii* genome, but we found more in the present study (Supplementary Table S6). In addition, our newly identified genes included genes involved in the cell cycle, e.g., cell division control protein 2-like (*CDC2*), proliferating cell nuclear antigen (*PCNA*), cyclin-L1-1, anaphase-promoting complex subunit, and growth arrest-specific protein 8-like (Supplementary Table S7).

### 3.3. Core Gene Set Refinement

It has been postulated that highly and commonly expressed genes under different growth conditions can be classified as core genes, whereas genes showing variable expression levels in response to growth condition changes are environment responsive genes [33]. In this study, a total of 2113 annotated genes were qualified as core genes based on their high and stable expression levels under five trace metal deficiencies and nutrient replete conditions, which we designated as core gene set 1 (Supplementary Table S8). The average expression of the core genes was 117.30 TPM, the lowest expression was 49.13 TPM, while the highest expression was up to 1855.28 TPM, but each specific gene displayed a relatively stable expression level under different growth conditions. Among the annotatable core genes, the six most highly expressed genes encode two light-harvesting proteins, two peridinin chlorophyll-*a* binding proteins (PCP), a hypothetical protein (Ctob_001144), and the translation elongation factor 1 alpha-like protein. Most of these genes are important in light harvesting, and have been reported as highly expressed genes in dinoflagellates [47], indicating that *F. kawagutii* has a high and stable capacity of light harvesting. Coral bleaching has been associated with the loss of light-harvesting protein under combined light and temperature stress [48]. *F. kawagutii* is known to hold a stable growth rate and maximum quantum yield of PSII (Fv/Fm) under high-temperature conditions [13,14], probably due to the high and stable ability of light harvesting.

**Figure 3:**
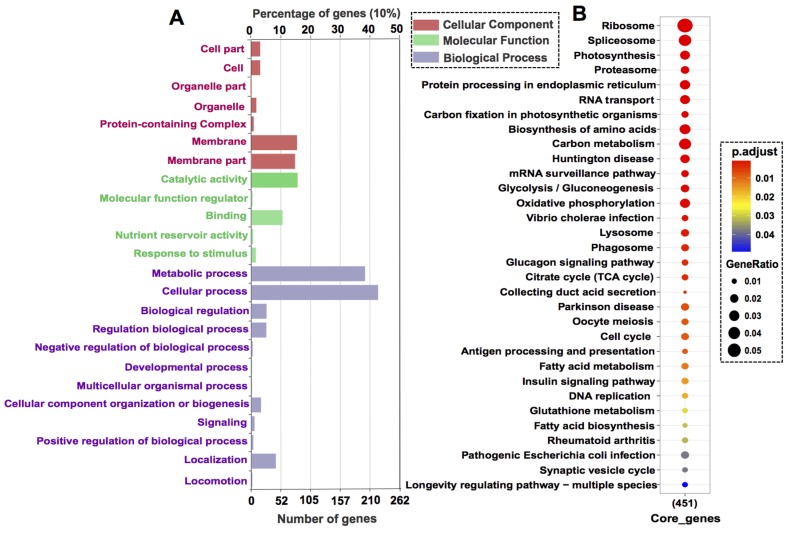
GO annotation and KEGG pathway enrichment of core genes: (**A**) GO annotation category (level 1 Go terms). (**B**) KEGG pathway enrichment with the *p*-value cutoff = 0.05. Dot size represents enriched DEGs count; color strength represents the *p*-value (from lowest in red to highest in blue).

Functions of the core genes were revealed by GO annotation and KEGG enrichment. The GO annotatable genes (525) were distributed in seven subcategories of cellular component, five subcategories in molecular function, and twelve subcategories in biological process (Figure 3A). The most highly enriched sub-categories are metabolic process and cellular process. The KEGG annotatable genes (451) were significantly enriched in thirteen 32 KEGG pathways (*p*-value cutoff = 0.05) such as ribosome, RNA transport, DNA replication, and cell cycle (Figure 3B).

We have previously identified 221 core genes in *F. kawagutii* exhibiting stable expression under heat stress, phosphate deprivation, glycerol-3-phosphate replacement, and nutrient replete conditions based on Fugka_Geneset_V1 as the reference gene set [33]. Among the 221 core genes, the two most highly and commonly expressed ones were 14-3-3 protein and ADP ribosylation factor, which are different from that found in the present study. Here, we combined these two versions of core gene sets by using BLASTN (*E*-value ≤ 10^−5^) and found 115 common core genes, generating core gene set version 2 (Supplementary Table S9). These 115 core genes were highly and commonly expressed under 9 treatment conditions. Among the 115 core genes, the five most highly expressed genes were hypothetical protein AK812_SmicGene36000, Major basic nuclear protein 2, Photosystem II 12 kDa extrinsic protein (chloroplastic), putative peptidase C1-like protein, and tubulin alpha chain.

### 3.4. Validation of Reference Genes for Gene Expression Studies

To accurately characterize the expression levels of a gene of interest, proper reference genes for normalization are essential. To date, only a few stably expressed reference genes have been identified in Symbiodiniaceae, such as genes encoding glyceraldehyde-3-phosphate dehydrogenase (*GAPDH*), ribosomal proteins, tubulin, translation elongation factor, aldolase A, and fatty acid desaturases identified for *Cladocopium* spp. (formerly *Symbiodinicum* ITS-type C_3_) [49]. Sometimes reference genes are only suited to specific species or treatment conditions. For instance, tubulin gene (*TUB*)was ranked as a better reference gene than S-adenosyl methionine synthetase (*SAM*) and calmodulin (*CAL*) under 25 °C and 33 °C in *Breviolum* spp. [50], but were shown to be less stable genes than *SAM* and *CAL* under thermal and light stress conditions in *Cladocopium* spp. [51]. Therefore, more candidate reference genes are needed. The core gene set identified in this study provides more candidate reference genes to fulfill this need for *F. kawagutii* (Supplementary Tables S8 and S9).

**Figure 4:**
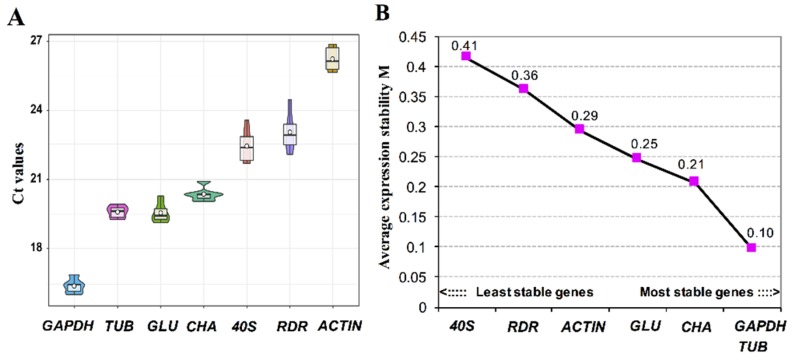
Housekeeping gene expression in nutrient-replete and +1/5Fe, –Mn, +1/5Zn, –Cu, –Ni deficient conditions: (**A**) Boxes of seven housekeeping gene expressions. (**B**) The stability and ranking of the selected genes shown from A calculated by geNorm. *TUB*: tubulin; *GLU*: beta-glucanase; *CHA*: chaperone protein; *40S*: 40S ribosomal protein S4; *RDR*: ribonucleoside-diphosphate reductase.

From the updated candidate reference gene set, seven genes that show greatest promise due to high and stable expression levels were selected for further analysis using RT-qPCR. As a result, the threshold cycle (*Ct*) ranged from 16.48 to 26.12, and for three of them, the value ranged from 19.54 to 20.35 (Figure 4A). The *GAPDH* showed the highest expression level with *Ct* value ranging from 16.04 to 16.90 under different trace metal deficiency conditions, with *ACTIN* exhibiting the lowest expression level with *Ct* value ranging from 25.53 to 26.75. To further rank the stability of expression level among the candidate reference genes, average expression stability value (M) was calculated using geNorm (Figure 4B). All the seven genes investigated in this study showed M values far smaller than the threshold value 1.5, indicating that the expression levels of these genes were relatively stable under trace metal deficiencies and the nutrient replete condition. Comparative analyses showed that the most stable genes were *TUB* and *GAPDH* (M = 0.10), and the 40S ribosomal protein S4 gene was the least stably expressed under trace metal deficiencies (Figure 4B). This is ironic because ribosomal protein encoding genes have been shown to be a suitable reference gene in RT-qPCR experiments in both animals and algae [49,51,52], and 40S ribosomal protein S4 gene was previously shown to be the most stably expressed among the nine selected candidate reference genes that were examined under thermal stress in *Cladocopium* spp. [51]. Based on our results, there are two possible explanations for the contradiction. Firstly, the 40S ribosomal protein S4 gene was strongly regulated specifically by trace metal availability. Secondly, 40S ribosomal protein S4 has different expression patterns in different species. Similarly, even though *GAPDH* has been considered a suitable reference gene in many algal species, such as *Prorocentrum donghaiense* [53], *Alexandrium catenella* [54], *Amphidinium carterae* [55], *Emiliania huxleyi* [34], and *Chlamydomonas* sp. [51,56], several reports have shown that its expression is not stable across different environment conditions [57,58,59]. Taken together, our data along with the previous data of others suggest that it is crucial to analyze the suitability of the reference gene(s) for different species or specific types of conditions before use in gene expression analysis.

**Figure A1:**
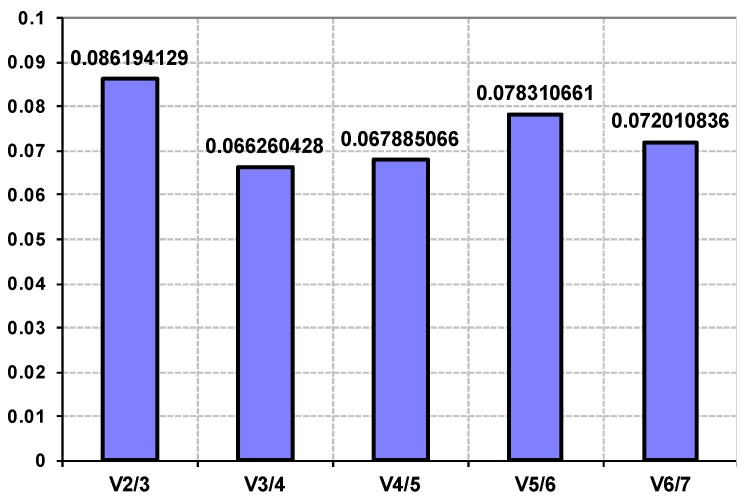
Comparison of multi-gene combinations to determine the optimal number of reference genes required for effective normalizing gene expression. The pairwise variation (Vn/Vn + 1, where *n* represent number of genes) between the normalization factors NF_n_ and NF_n + 1_ was analyzed using geNorm. All the combinations gave a normalization factor value below the upper threshold 0.15, indicating that two-gene combinations are sufficient.

The geNorm analysis was also conducted to determine the optimal number of reference genes required for use in combination for effective normalization, based on the pairwise variation (V_n_/V_n + 1_) value [35]. In the present study, V2/3 score was 0.086, far lower than the threshold of 0.15, indicating that the combination of two reference genes is sufficient for normalizing gene expression under trace metal deficiencies and nutrient replete conditions (Appendix A Figure A1).

## 4. Conclusions

Our integrative Hi-C and deep transcriptomics sequencing using a combination of Illumina and PacBio technologies resulted in a new version of *F. kawagutii* genome and proteogenome (Fugka_Geneset_V3), and our analysis indicates that this gene set represents a significant improvement from the previous two versions of the *F. kawagutii* genome. This gene set contains 11,984 functionally additional genes compared to the previous versions of the genome, which are significantly enriched in 38 KEGG pathways. This brings the total gene number up to 45,192 in this species. Our analysis in this study also reveals 2,113 annotatable genes that were stably expressed under different trace metal conditions and 115 genes that were commonly stably expressed under nine treatment conditions, leading to the refinement of the “core gene set” and addition to candidate reference genes repertoire. The *GAPDH* and *TUB* were found to be the most stable reference genes for trace metal stress studies and the combination of two reference genes found to be sufficient for normalizing gene expression under trace metal deficiencies. The improved gene set led to an increase of transcriptomic read mapping rate from 33% to 54%, and a BUSCO match from 29% to 55%. The improved version of the genome data will prove useful for future transcriptomics, gene structures, and gene expression studies, and the updated core gene set provides abundant candidates for identifying reference genes for gene expression studies.

## Figures and Tables

**Table 1:** Sequenced genomes of Symbiodiniaceae.

Species	Assembled Genome Size (M)	% Genome Assembled	Gene No.	Scaffold N50 (kbp)	Gene Average Length (+ Intron)	Gene Supported by EST *(%)	Reference
***B. minutum***	616	41.07	41,925	126.2	11,959	77.20	[8]
***S. microadriacticum***	808	73.45/57.71	49,109	573.5	12,898	76.30	[11]
***C. goreaui***	1030	85.55	35,913	98.9	6967	67.02	[10]
***Symbiodinium* spp.**	767	NA	69,018	133.4	8834	67.50	[12]
***Cladocopium* spp.**	705	NA	65,850	248.9	8192	62.50	[12]
***F. kawagutii***	935	79.24	36,850	381	3788	72.82	[9]
	1050	88.98	26,609	268.8	6507	64.40	[10]
	937	79.41	45,192	13,533.5	7242	90.09	This study

* EST: Expressed Sequence Tag; NA: Not available.

**Table 2:** RNA-Seq sample information of *F. kawagutii*.

Project	Methodology	Growth Condition	Clean Read Data	Mapping Rate (Genome, Geneset)		
				V1 *	V2 **	V3 ***
**Transcriptome1 ^#^**	BGI RNA-seq (DGE) (SE 50)	Normal Low Cu, Zn, Fe, Mn, Ni	329 M	75%, 33%	69%, 33%	69%, 54%
**Transcriptome2 ^#^**	Illumina NGS (PE150)	Mix of 1^#^	10 Gbp	88%, 25%	87%, 29%	87%, 50%
**Transcriptome3 ^#^**	Pacbio Sequel (full-length)	Mix of 1^#^	15 Gbp	99%, NA	99%, NA	99%, NA

V1 *: Fugka_Geneset_V1 (first version of the genome, [9]); V2 **: Fugka_Geneset_V2 (second revision of the genome, [10]); V3 ***: Fugka_Geneset_V3 (third revision of the genome, This study); NA: Not available.

**Table 3:** BUSCO (429 orthologs protein) evaluation of gene set completeness.

BUSCO	V1	V2	V3
**Complete**	68	126	141
**Complete and single-copy**	56	112	130
**Complete and duplicated**	12	14	11
**Fragmented**	57	67	92
**Missing**	304	236	196
**Total detected (%)**	29%	45%	55%

## Supplementary Materials

The following are available online at https://www.mdpi.com/2076-2607/8/1/102/s1.
